# Potential dermal wound healing agent in *Blechnum orientale *Linn

**DOI:** 10.1186/1472-6882-11-62

**Published:** 2011-08-12

**Authors:** How Yee Lai, Yau Yan Lim, Kah Hwi Kim

**Affiliations:** 1 School of Biosciences, Taylor's University Lakeside Campus, No. 1, Jalan Taylor's, 47500 Subang Jaya, Selangor, Malaysia; 2 School of Science, Monash University Sunway Campus, Bandar Sunway, 46150 Petaling Jaya, Selangor, Malaysia; 3 Department of Physiology, Faculty of Medicine, University of Malaya, 50603 Kuala Lumpur, Malaysia

## Abstract

**Background:**

*Blechnum orientale *Linn. (*Blechnaceae*) is used ethnomedicinally to treat wounds, boils, blisters or abscesses and sores, stomach pain and urinary bladder complaints. The aim of the study was to validate the ethnotherapeutic claim and to evaluate the effects of *B. orientale *water extract on wound healing activity.

**Methods:**

Water extract of *B. orientale *was used. Excision wound healing activity was examined on Sprague-Dawley rats, dressed with 1% and 2% of the water extract. Control groups were dressed with the base cream (vehicle group, negative control) and 10% povidone-iodine (positive control) respectively. Healing was assessed based on contraction of wound size, mean epithelisation time, hydroxyproline content and histopathological examinations. Statistical analyses were performed using one way ANOVA followed by Tukey HSD test.

**Results:**

Wound healing study revealed significant reduction in wound size and mean epithelisation time, and higher collagen synthesis in the 2% extract-treated group compared to the vehicle group. These findings were supported by histolopathological examinations of healed wound sections which showed greater tissue regeneration, more fibroblasts and angiogenesis in the 2% extract-treated group.

**Conclusions:**

The ethnotherapeutic use of this fern is validated. The water extract of *B. orientale *is a potential candidate for the treatment of dermal wounds. Synergistic effects of both strong antioxidant and antibacterial activities in the extract are deduced to have accelerated the wound repair at the proliferative phase of the healing process.

## Background

*Blechnum orientale *Linn. (*Blechnaceae*) is a perennial terrestrial fern natively distributed in Malaysia and is commonly known as the Centipede fern or 'paku ikan' (by the Malays) or 'dungau' (by natives in Sabah) or 'Kuan Chung' (by the Chinese). It is used ethnomedicinally to treat wounds, boils, blisters or abscesses and sores [1], for stomach pain, urinary bladder complaints [2,3] and sterilization of women [4]. Young fronds are boiled and eaten as vegetable by the natives [5,6]. As its ethnotherapeutic use in wound treatment has not been scientifically researched and in view of the strong bactericidal and antioxidant activities of the water extract as reported in our previous studies [7,8], an *in vivo *investigation was undertaken to evaluate the potential of the extract for treatment of wounds.

Wound healing is currently a clinical challenge due to inconsistencies encountered in the healing processes. Medical treatment includes administration of drugs either locally (topical) or systemically (oral or parenteral) with the aim to either shorten the time required for healing or to minimize the undesired consequences during wound repair [9]. Medicinal plants have generated much interest for treatment of skin ailments as they are affordable and purportedly safe from hypersensitive reactions [10].

Wound is defined as a breaking of cellular and anatomical or functional continuity of living tissue [11]. There are three phases in the process of wound healing. Phase 1 is the coagulation and inflammatory phase that involves migration of neutrophils at the margin of incision. Phase 2 is the proliferative phase which is characterized by angiogenesis, collagen deposition, epithelization and wound contraction. Angiogenesis involves new blood vessel growth from endothelial cells. Granulation tissue progressively invades the incision space. Collagen fibrils become more abundant and begin to bridge the incision. At this phase, the epithelization depends on the migratory, proliferative and differential abilities of keratinocytes and these are regulated by growth factors such as epidermal growth factor family and fibroblast growth factor family [12,13]. Phase 3 is a remodeling phase involving continuous accumulation of collagen and proliferation of fibroblasts. This phase involves synthesis of collagen fibers, leading to increase in tensile strength of the skin [14]. Alterations in any of these steps can lead to healing delay or even the inability to heal completely [15].

Our earlier studies [7,8] have revealed the presence of tannins in the water extract. This extract was also found to possess strong antioxidant and antibacterial activities. The radical scavenging activity of the water extract was found to be equivalent to the reference α-tocopherol with IC_50 _13.0 ± 1.3 μg/mL [7]. The water extract also recorded good bactericidal activities against five Gram-positive bacteria including methicillin-resistant *Staphylococcus aureus *MRSA (minimum bactericidal concentration MBC 62.5 μg/mL) [7]. Subsequently, we undertake this study to support our hypothesis that both antioxidant and antibacterial activities in the water extract could play a synergistic role in the treatment of wounds and also to validate scientifically its ethnotherapeutic role in skin diseases. To the best of our knowledge, this is the first report on the efficacy of *B. orientale *on wound healing.

## Methods

### Plant material and extraction

*Blechnum orientale *Linn. was obtained from Putrajaya Botanical Garden, Kuala Lumpur. The identity was confirmed by plant taxonomist Anthonysamy S., formerly from University Putra Malaysia and currently a consultant with the landscape consulting firm, Aroma Tropic Limited, Kuala Lumpur. A voucher specimen (LAA007) was deposited at the Herbarium of Monash University Sunway Campus.

The extracts were prepared as previously described [7]. Briefly, powdered leaves of *B. orientale *were extracted with methanol at room temperature. The extract solution was filtered and the solvent was evaporated under reduced pressure. After freeze-dried, the dark green mass obtained (17% yield based on dry leaves) was suspended in distilled water (1:10, w/v) and partitioned successively with petroleum ether 40-60°C, chloroform, ethyl acetate and *n*-butanol. In the final partitioning with butanol, the lower water layer was removed and concentrated under reduced pressure. It was then freeze-dried to obtain a dry brown powder mass labelled as the water extract (6.5% yield based on dry leaves). The sample was stored at -70°C until used.

### Quantitation of total tannins

Total tannins were determined as previously described [16]. Tannins were distinguished from nontannins by using polyvinylpolypyrrolidone (PVPP) which has a high affinity for tannins. Total phenolics content (TPC) was measured using the Folin-Ciocalteau method [7] before and after treatment with PVPP. Treatment with PVPP was conducted as follows. Distilled water (1 mL) was added to PVPP (100 mg) before adding 1 mL extract. The mixture was vortexed and centrifuged at 3000 g for 10 min. Supernatant was collected and TPC was determined as before. The standard curve was prepared using 2 - 10 μg/mL tannic acid. Tannin content was calculated as the difference between total phenolics (before PVPP treatment) and the nontannin phenolics (after PVPP treatment). Results were expressed as grams tannic acid equivalent (TAE) per 100 g dry weight.

### Animals model

This study was approved by the University Ethics Committee of the Monash University for animal experimentation (SOBSB/MY/2009/46). Sprague-Dawley rats (200 - 250 g) of either sex were purchased from the Animal House of Monash University Sunway campus. Each animal was caged individually and acclimatized for 7 days, under a climate-controlled environment (22.0 ± 3°C) and relative humidity 30-70%), 12-h dark and light cycles. Standard rodent chow pellets were given *ad libitum *with free access to water.

### Materials

Aqueous cream (manufactured by Pharmaniaga Manufacturing Bhd, Malaysia) and povidone-iodine 10% solution (manufactured by Polylab Sdn. Bhd, Malaysia) were purchased from a local pharmacy store. 4-dimethylaminobenzaldehyde and chloramines T were purchased from Acros, citric acid and sodium citrate from Fischer and hydroxyproline from Sigma. All other chemicals were of extra-pure grade and used as received.

Two concentrations of the water extract cream was formulated using aqueous cream base as the vehicle. The aqueous cream consisted of emulsifying wax (9%), white soft paraffin (15%), liquid paraffin (6%), chlorocresol (0.1%), glycerin (5%) and purified water. For 1% (w/w) extract cream, 1 g of the dry water extract was incorporated in 100 g of aqueous cream and warmed at 50-55°C, with constant stirring until a homogeneous extract-cream formation was obtained. For 2% (w/w) extract cream, 2 g of the dry water extract was used in place of 1 g of the extract. The extract cream was weighed into eppendorf tubes (approximaely 0.20 g per tube) and left to equilibriate at room temperature for 3 days, before use.

### Wound healing activity

The procedure described by Nayak *et al*. (2009) [13] was followed with slight modifications. The animals were divided into four groups with six animals per group. Animals were anaesthetized by intraperitoneal injection of ketamine/xylazine (ketamine at 100 mg/kg and xylazine 10 mg/kg). An area (150 mm^2^) was marked using a frame and marker pen. The required area (approximately 5 mm bigger than the marked area) of the dorsal fur of the animals was shaved with an electric clipper. The area was sterilized by spraying with 70% ethanol. A full thickness skin (150 mm^2^) was excised from the predetermined area by removing the epidermis and dermis layer until the subcutaneous fat (avoiding panniculus carnosus and the muscle layer). Carprofen at 5 mg/kg was injected subcutaneously every day for 5 days as analgesia.

Group I was applied topically with aqueous cream (negative control), group II with povidone-iodine 10% (Polylab^®^, positive control), groups III and IV with 1% and 2% (w/w) water extract cream respectively. The reference (povidone-iodine), extract cream and the base cream were applied topically (dose approximately 0.20 g/wound) once a day until the wound was completely healed or to a maximum of 14 days. Special care was taken to avoid variation in the dose given.

Animals were monitored every day. An animal monitoring sheet was used to record all observations e.g. its activity, alertness, body condition, body weight, breathing, its coat condition, signs of dehydration, drinking, eating, conditions of its eyes, feces, nose, urine, its movement and vocalization.

The wound area contractions were measured on the 1^st ^(wounding day) and thereafter every alternate day until completely healed. The wound margin was traced on a sterile autoclaved transparent paper (3 times to get an average area) and then placed on a graph paper to determine the area. Wound contraction was calculated as percentage reduction of initial wound area. Wounds were considered closed (completely healed) if moist granulation tissue was no longer apparent and the wound was covered with new epithelium.

After complete healing, rats were killed using carbon dioxide gas [17]. The healed skin was excised. A small piece of tissue was fixed in 10% formalin for histopathological examination. The remaining tissue was used for the determination of total collagen in the hydroxyproline assay.

### Determination of total collagen - Hydroxyproline assay

The procedure used for the hydrolysis of the granulation tissue is as described by Nayak *et al*. (2009) [13]. The wet weight of the granulation tissue was recorded. The tissue was dried at 60°C for 12 h and the dry tissue weight recorded. To the dried tissue, 5 mL 6 N HCl was added and autoclaved at 120°C for 20 min. The neutralized acid hydrolysate of the dry tissue was used for the hydroxyproline assay.

Total collagen was determined following the method described by Jorge *et al*. (2008) [14]. Hydrolyzed samples (20 μL) were added to 96-well plate and incubated for 20 min at room temperature with 50 μL/well of chloramines T solution (282 mg chloramines T, 2 mL *n*-propanol, 2 mL distilled water, and 16 mL citrate acetate buffer). Then 50 μL/well of Erlich's solution (2.5 g 4-dimethylamino benzaldehyde, 9.3 mL *n*-propanol, and 3.9 mL 70% perchloric acid) was added and incubated for 15 min at 65°C. Absorbance was measured at 550 nm with a microplate reader. Hydroxyproline concentrations from 0 to 20 μg/mL were used to make a standard curve. Results were expressed as mg of hydroxyproline per g of dry tissue.

### Histopathological studies

Skin specimens were immediately fixed in 10% (v/v) neutral-buffered formalin with the fixative solution replaced every 2 days until the tissues hardened. Each specimen was embedded in a paraffin block and thin sections (3 μm) were prepared and stained with Masson trichrome (for detection of collagen fibers) and haematoxylin and eosin (H&E) (for general morphological observations). Slides were examined qualitatively under a light microscope, for collagen formation, fibroblast proliferation, angiogenesis, epithelization and granulation tissue formation, employing light to intense scale (+ to +++) [15,18].

### Statistical analysis

All data were expressed as mean ± SD. Statistical analyses were evaluated by one-way ANOVA followed by Tukey HSD test. Values with P < 0.001 were considered statistically significant.

## Results

Previous studies [7] have shown that the water extract of *B. orientale *consisted of tannins and possessed strong DPPH radical scavenging (IC_50 _13.0 ± 1.3 μg/mL) and antibacterial activities (minimum inhibitory concentrations towards MRSA, MSSA, *M. luteus*, *B. cereus *and *S. epidermidis *were 31.3 - 62.5 μg/mL) (Table 1). In this study, total tannins in the water extract were found to be 20 ± 4% (g tannic acid equivalent/100 g extract).

**Table 1:** Antioxidant and antibacterial activities of water extract of *B. orientale *[7]

	Water extract of *B. orientale*	Reference used
DPPH radical scavenging activity (IC_50_, μg/mL)	13.0 ± 1.3	12.0 ± 0.7 (α-Tocopherol)

Antibacterial activities (Minimum inhibitory concentration MIC, μg/mL)	MRSA 62.5 MSSA 62.5 *M. luteus *31.3 *B. cereus *62.5 *S. epidermidis *62.5	1.9 (Vancomycin) 1.9 (Vancomycin) 1.9 (Vancomycin) 1.9 (Vancomycin) 1.9 (Vancomycin)

Abbreviation: DPPH, 1,1-diphenyl-2-picrylhydrazyl. Microorganisms: MRSA, methicillin-resistant *Staphylococcus aureus *ATCC33591; MSSA, methicillin-susceptible *Staphylococcus aureus *ATCC25923; *M. luteus*, *Micrococcus luteus *ATCC4698; *B. cereus*, *Bacillus cereus *ATCC14579; *S. epidermidis*, *Stapylococcus epidermidis *ATCC12228.

### Wound contraction and days of epithelization

**Figure 1:**
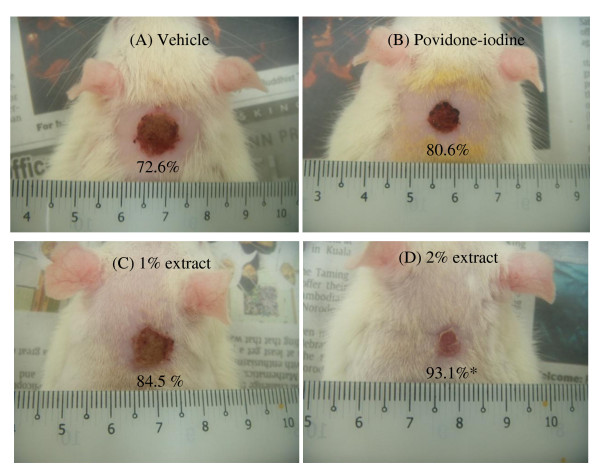
**Appearance of wounds at Day 10 post wounding**. A = vehicle group, B = povidone-iodine treated group, C = 1% extract-treated group, D = 2% extract-treated group. Values are mean ± S.D. for 6 rats, showing % contraction of wound size on Day 10. * implies significant difference (P < 0.001) in comparison with the vehicle group.

Results of the wound contraction and days of epithelization are shown in Table 2. The 2% extract-treated group demonstrated significantly higher wound contracting ability (P < 0.001) than the vehicle group. The wound area of the 2% extract-treated group, as measured on every alternate day, showed significant contraction from 20% on day 2, to 62% on day 6, 93% on day 10 (Figure 1D) and was completely healed on day 14. In comparison, vehicle group showed only 4% contraction on day 2, 41% on day 6, 73% on day 10 (Figure 1A) and 85% on day 14 (Table 2). It is of interest to note that similar contractions were seen in both 1% extract-treated and povidone-iodine groups e.g. on day 2 (16-18%), day 4 (28-30%), day 8 (75-77%), day 10 (81-85%), day 12 (90-93%) and day 14 (95-97%) (Table 2). Overall, the contraction of wound was in the order of 2% extract-treated > 1% extract-treated ≈ povidone-iodine-treated > vehicle.

**Table 2:** Effect of extracts on wound contraction and days of epithelization

Day	**Wound area (mm**^**2**^**) ± S.D. (% contraction)**			
	Vehicle	Povidone-iodine	1% extract	2% extract
0	169.8 ± 13.8	162.2 ± 9.5	165.2 ± 15.2	160.2 ± 17.8
2	163.7 ± 10.4 (4%)	133.7 ± 12.0 (18%)	139.7 ± 15.4 (16%)	128.7 ± 13.4 (20%)*
4	132.6 ± 11.9 (22%)	116.0 ± 10.9 (28%)	115.8 ± 7.1 (30%)	101.0 ± 10.7 (37%)*
6	100.0 ± 18.7 (41%)	92.0 ± 12.2 (44%)	71.0 ± 6.7 (57%)	60.5 ± 4.7 (62%)*
8	54.3 ± 9.8 (67%)	40.8 ± 8.3 (75%)	38.1 ± 1.8 (77%)	25.6 ± 1.8 (84%)*
10	45.8 ± 5.5 (73%)	30.8 ± 7.5 (81%)	25.5 ± 2.0 (85%)	13.0 ± 1.5 (93%)*
12	32.0 ± 5.9 (81%)	11.5 ± 5.9 (93%)	16.5 ± 2.9 (90%)	4.9 ± 0.6 (97%)*
14	25.5 ± 6.4 (85%)	5.0 ± 0.9 (97%)	8.8 ± 0.9 (95%)	0.8 ± 0.9 (100%)*

Days of epithelization	19.2 ± 1.5	16.6 ± 1.0	17.6 ± 0.9	13.0 ± 0.4*

Results are as mean ± S.D. for 6 rats per group. Percentages of contraction are recorded in parentheses. * implies significant difference at P < 0.001 compared to the vehicle group.

Epithelization time refers to the number of days taken by the wounds to appear completely closed with no moist granulation tissue and the wound was covered with new epithelium. Wounds dressed with 2% extract were found to epithelize the fastest (13.0 days), followed by povidone-iodine group (16.6 days) ≈ 1% extract group (17.6 days) while the vehicle group took an average of 19.2 days to completely heal (Table 2). Nevertheless, there were no significant differences in the mean epitheliziton time among wounds dressed with 1% extract, vehicle and povidone-iodine. This indicated the healing potential of the extracts was dose dependent and was effective only at a 2% concentration of the extract.

### Collagen synthesis

**Figure 2:**
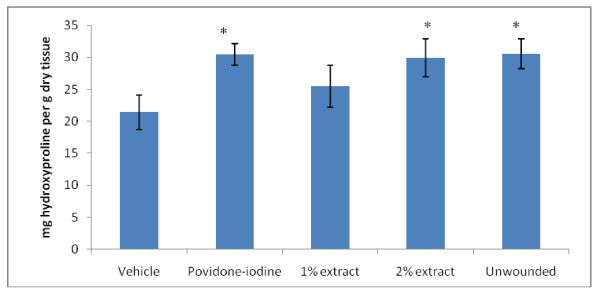
**Effect of extracts on *in vitro *collagen synthesis as measured by hydroxyproline analyses**. Data are mean ± S.D of triplicate measurements from 6 rats. *implies significant difference at p < 0.001 compared to the vehicle group.

Hydroxyproline is an amino acid essential for collagen synthesis. For this reason, hydroxyproline content has been used as a marker to determine the collagen content [19]. Estimation of hydroxyproline content revealed significantly higher hydroxyproline content in the 2% extract-treated animals, povidone-iodine control group and the normal (unwounded) skin compared to that of the vehicle (Figure 2). Nevertheless, there were no significant differences in the level of hydroxyproline when the animals were treated with 1% or 2% of the extract.

### Histopathological study

**Figure 3:**
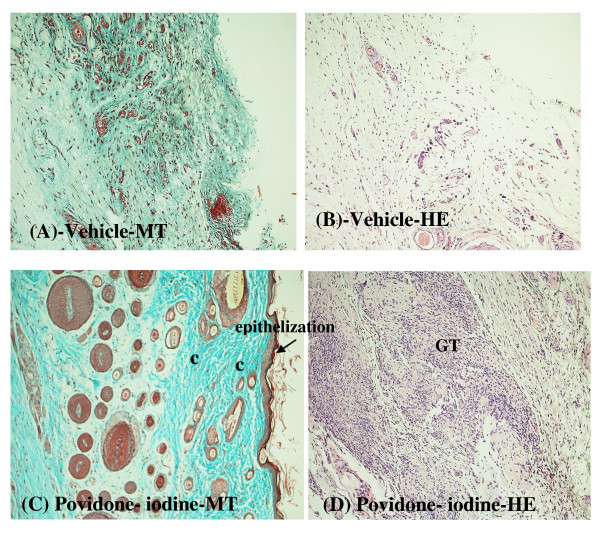
**Histological examination of healed wound sections of the vehicle (A and B) and povidone-iodine-treated group (C and D)**. MT refers to Mason trichrome stained; HE refers to hematoxylin-eosin stained. Magnification: 100×. Abbreviation: c, collagen fibers; GT, granulation tissue; bc, blood capillaries.

**Figure 4:**
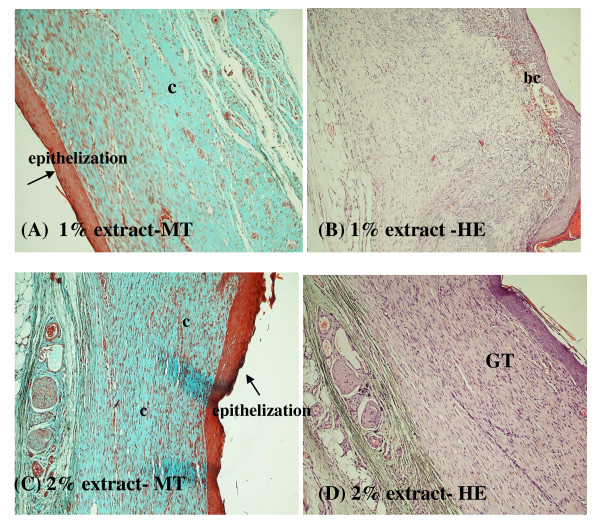
**Histological examination of healed wound sections of 1% extract-treated group (A and B) and 2% extracted treated group (C and D)**. MT refers to Mason trichrome stained; HE refers to hematoxylin-eosin stained. Magnification: 100×. Abbreviation: c, collagen fibers; GT, granulation tissue; bc, blood capillaries.

Histopathological examinations of the healed wounds are shown in Figures 3 and 4. Two types of stains were used: Masson-trichrome (MT) for collagen deposition and hematoxylin & eosin (H&E) for general morphology. Masson-trichrome stains collagen blue, while cytoplasm, red blood cells and muscle are stained red, and is typically used to assess the advancement of collagen deposition during the formation of granulation tissue and matrix remodeling [20]. The blue colour staining intensity corresponds to the relative quantity of collagen fiber deposit, which reflects the process of synthesis and degradation and remodeling as well as the timing of the lesion [21]. H&E stains collagen fibers pale pink, cytoplasm purple, nuclei blue and red blood cells cherry red.

**Figure 5:**
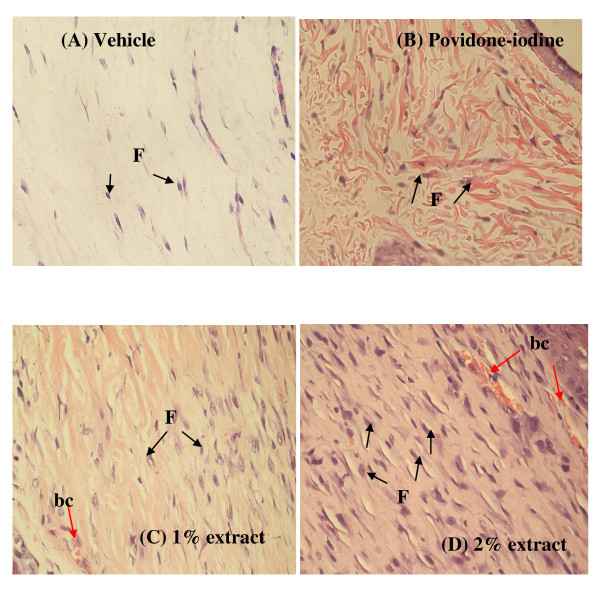
**Histological examination of healed wound sections stained with hematoxylin-eosin (HE, 400× magnification)**. Abbreviation: bc, blood capillaries; F, fibroblast. Black arrow points to fibroblast cells; red arrow points to bc.

Greater tissue regeneration was observed in the povidone-iodine-treated group (Figure 3C and 3D) and the 2% extract-treated group (Figure 4C and 4D) as demonstrated by the complete epithelization (Figure 3C and 4C), significantly higher collagen deposition (more intense blue coloration in Figure 3C and 4C) and granulation tissues (Figure 3D and 4D) compared to the healed wounds dressed with vehicle (Figure 3A and 3B). Figure 5 shows the histological patterns of the sections stained in H&E at 400× magnification. More number of fibroblast cells and newly formed blood capillaries (angiogenesis) were observed with the 2% extract-treated group (Figure 5D) in comparison to the vehicle group (Figure 5A) and 1% extract-treated group (Figure 5C). These observations indicated a dose-dependent response in wound healing activity (Figure 5C and 5D).

## Discussion

Our histological findings correlated well with the findings from wound contraction measurement, mean epithelization time study and the biochemical marker test of the hydroxyproline. The significant reduction in wound size and mean epithelization time as well as the higher hydroxyproline content in the 2% extract-treated group as compared to those from vehicle group corroborates with the histopathological findings of increased epithelization activity, angiogenesis, granulation tissue formation and higher collagen fibers formation (Figures 3, 4, 5 and Table 3). These findings imply that the tannins present in the water extract of *B. orientale *promoted wound healing activity by acting at the proliferative stage via angiogenesis, collagen deposition, granulation tissue formation, epithelization and wound contraction.

**Table 3:** Histopathological evaluation of wound healing parameters on healed wounds of the vehicle-, povidone-iodine-, 1% extract- and 2% extract-treated animals

Groups	Collagen formation	Fibroblast proliferation	Angiogenesis	Epithelization
Vehicle	+	+	++	+
Povidone-iodine	+++	+++	++	+++
1% extract	++	++	++	++
2% extract	+++	+++	+++	+++

Sections were scored from light (+), moderate (++) or intense (+++) based on relative intensity of the blue coloration in the MT stained sections (for collagen evaluation), the relative density of fibroblast cells and blood capillaries in the H&E stained sections (for fibroblast proliferation and angiogenesis) and the complete formation of epidermis in the H&E stained sections (for epithelization).

Polyphenolic flavonoids and tannins are reported to facilitate wound healing [11]. The wound healing potential of the water extracts of *B. orientale *is a result of the mixture of these phyto-constituents as substantial amounts of these have been found in our earlier study [7]. Tannins promote wound healing through several cellular mechanisms: scavenging of free radicals and reactive oxygen species, promoting contraction of the wound and increasing the formation of capillary vessels and fibroblasts [22]. A quantitative analysis on the tannin content of the water extract revealed 20 ± 4% tannins (measured as g tannic acid equivalent/100 g extract). Similar findings have been reported with extracts of plants containing 20-40% tannins [22,23]. The astringent and antimicrobial properties of tannins are important attributes to the wound healing properties [22].

Our earlier studies have revealed strong antioxidant activity (comparable to α-tocopherol) and bactericidal activity against methicillin-resistant *Staphylococcus aureus *(MRSA), *Staphylococcus aureus*, *Micrococcus luteus*, *Bacillus cereus *and *Staphylococcus epidermidis *[7]. The ability to scavenge free radicals and exert bactericidal effect is known to play an important role in the treatment of wounds at the proliferative stage. Reactive oxygen species can induce severe tissue damage and even lead to neoplastic transformation decreasing the healing process by damages in cellular membranes, DNA, proteins and lipids [24]. Skin pathogens such as *S. aureus*, *S. epidermidis *and *M. luteus *are also important contributing factors for skin infections leading to inflammation and causing delay in wound healing processes. For example, curcumin isolated from *Curcuma longa *Linn. is reported to have both anti-inflammatory and wound healing activity through its antioxidant property [25], while manuka honey contributes to wound healing through its bactericidal activity against a host of skin pathogens including MRSA [26,27]. Hence, since the water extract possessed both antioxidant and antibacterial activities, the conjoint effects on wound healing processes render it a promising candidate for the treatment of wounds and this also justified its traditional usage in wound treatment.

## Conclusions

The results revealed a potential for the water extract of *B. orientale *to be used as an external application for the treatment of wounds. The water extract cream in 2% (w/w) concentration was capable of producing significant (p < 0.001) wound healing activity. Histopathological findings correlated well with wound contractions, mean epithelisation time study and the biochemical marker test of hydroxyproline. The mechanism of action of the extracts was postulated to be via angiogenesis, collagen deposition, granulation tissue formation, epithelization and wound contraction at the proliferative stage and these actions are attributed to the synergistic effects of the strong antioxidant and antibacterial effect of tannins in the extract.

## Competing interests

The authors declare that they have no competing interests.

## Authors' contributions

HYL carried out the experimentation as part of PhD study and drafted the manuscript. YYL supervised the work, evaluated the data and corrected the manuscript for publication. KHK supervised the work and evaluated the data. All authors read and approved the final manuscript.

## Pre-publication history

The pre-publication history for this paper can be accessed here:

http://www.biomedcentral.com/1472-6882/11/62/prepub
